# Supplementary material for: Transplantation of Chemical Compound-Induced Cells from Human Fibroblasts Improves Locomotor Recovery in a Spinal Cord Injury Rat Model
Source: Int J Mol Sci. 2023 Sep 8;24(18):13853. doi: 10.3390/ijms241813853 (PMC10530737; doi:10.3390/ijms241813853)
Supplement: Supplementary file 1 [file ijms-24-13853-s001.zip › ijms-2447964-supplementary.pdf]

Table S1. The primer sequences.

	Forward primer	Reverse primer
hTBP	GCCCGAAACGCCGAATATAATC	AAAATCAGTGCCGTGGTTCG
hHGF	AAACAATGCCTCTGGTTCCC	AGCTGCGTCCTTTACCAATG
hBDNF	TAACGGCGGCAGACAAAAAG	AACCCATGGGATTGCACTTG
hBMP2	AAACCTGCAACAGCCAACTC	AAACTTTCCCACCTGCTTGC
hBMP4	AACCAACCATGCCATTGTGC	ATGGAGATGGCACTCAGTTCAG
hIL-1RN	TCCTGTGTCAAGTCTGGTGATG	GCTTGTCCTGCTTTCTGTTCTC
hCXCL12	TCTTCGAAAGCCATGTTGCC	TTCGGGTCAATGCACACTTG

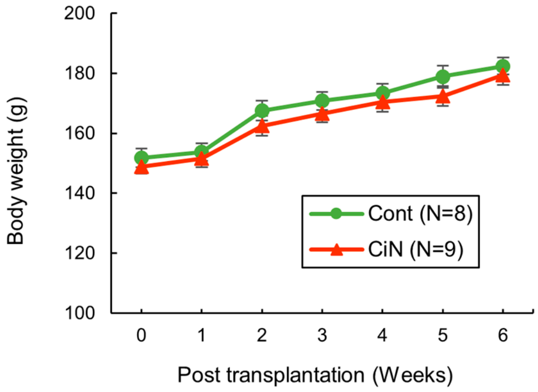

Figure S1. Body weight change in each group from the time of transplantation to the end of observation.

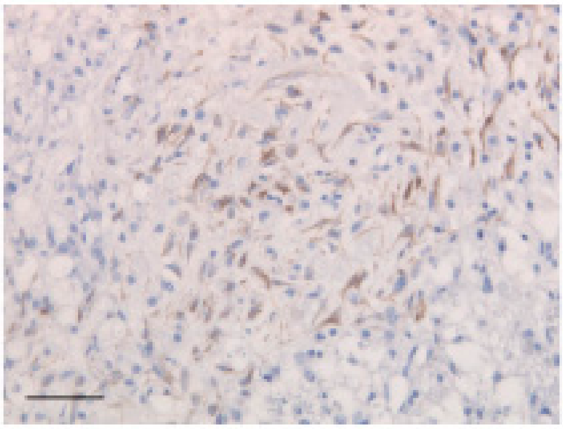

Figure S2. Detection of transplanted immature CiN cells at SCI site. The rats were dissected one week after the transplantation to confirm whether the transplanted cells engrafted at the SCI site. Immunohistochemical analysis was performed with an anti-STEM121 antibody, a human cytoplasmic marker. STEM121-positive staining was visualized using diaminobenzidine, which produces a brown reaction product. Cell nuclei were stained with hematoxylin (blue): scale bar, 50 μm.

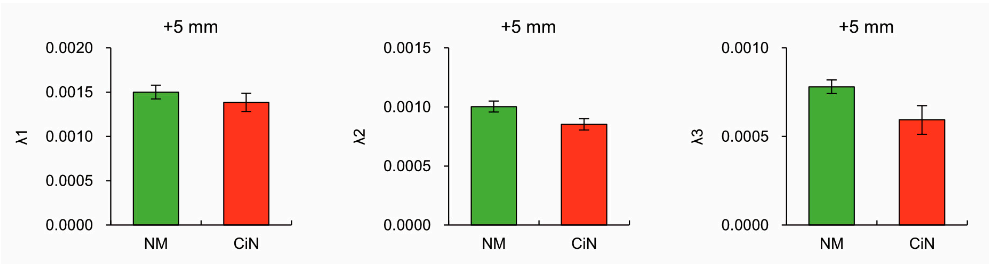

Figure S3. The eigenvalues of the MRI analysis. $\lambda1$, $\lambda2$, and $\lambda3$ are calculated by performing a diagonalization transformation on the tensor obtained by the MRI analysis.

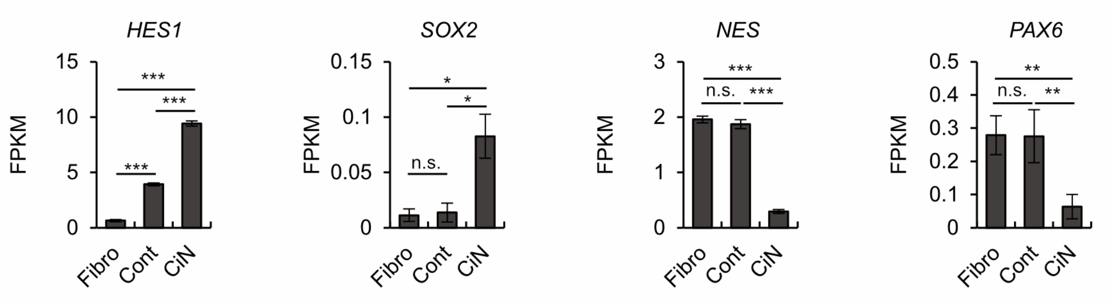

Figure S4. Transcript levels were quantified as fragments per kilobase of transcript per million mapped reads (FPKM). The FPKM of NSCs/NPCs marker genes such as HES1, SOX2, NES, and PAX6 were obtained from the RNA-seq results. The data are expressed as group means ± SEM (n = 3). A P-value of less than 0.05 was considered significant. *; $P < 0.05$, **; $P < 0.01$, ***; $P < 0.001$., n.s.; not significant.
